# Supplementary material for: Identification and functional analysis of non-coding regulatory small RNA FenSr3 in Bacillus amyloliquefaciens LPB-18
Source: PeerJ. 2023 May 15;11:e15236. doi: 10.7717/peerj.15236 (PMC10194069; doi:10.7717/peerj.15236)
Supplement: Supplemental Information 4 [file peerj-11-15236-s004.zip › KO/CK-vs-T1_map/map00966.html]

KEGG PATHWAY: Glucosinolate biosynthesis - Reference pathway


|  |  |
| --- | --- |
| **Glucosinolate biosynthesis - Reference pathway** |  |

[
Pathway menu
| Organism menu
| Pathway entry
| Show description
| User data mapping
]

Glucosinolates are biologically active secondary metabolites found in Brassicaceae (mustard family) and related families.These compounds are genetically variable within plant species and used as natural pesticides, such as against insect herbivores. All glucosinolates share a common structure consisting of a beta-thioglucose moiety, a sulfonated oxime moiety, and a variable aglycone side chain derived from an alpha-amino acid. Genes encoding glucosinolate biosynthetic enzymes have been identified in Arabidopsis thaliana by genetic polymorphisms and loss-of-function mutations. This map shows examples of side chain elongation in methionine-derived glucosinolates and the core pathway for biosynthesis of glucosinolates from amino acids.

|  |  |  |
| --- | --- | --- |
| Reference pathway | 184% 150% 122% 100% 82% 67% 55% | 图片下载 |
